# Fourteenth International Medical Congress

**Published:** 1903-09

**Authors:** Jefferson D. Griffith

**Affiliations:** Kansas City, Mo.; Grand Ave. and 35th Street


### Fourteenth International Medical Congress.

**From New York to Madrid and Return with Cook & Son.**

By JEFFERSON D. GRIFFITH, M.D., Kansas City, Mo.

ON the afternoon of April 11, 1903, at 4 o'clock, the magnificent North German Lloyd steamship, Princess Irene, started up her propellers, never missing a revolution until daylight of the 20th. After a remarkably smooth voyage Gibraltar was reached,—that British sentinel, harbor for battleships, gateway to and from the Mediterranean Sea,—through which Tarik, the Moor, in 711 introduced Arabian refinement into Europe.

GIBRALTAR.

This spur of land, running south from the Spanish peninsula, is within 20 miles of the African shore. The rock on the north and east rises almost perpendicularly 1,500 feet, is punctured with almost innumerable port-holes, and its summit bristles with guns, over which floats the English jack,—an impregnable fort that has withstood all attacks since its first capture in 1704.

To an outsider, this fort should belong to Spain. Great Britain, however, has expended 50 millions sterling on this station, and is now putting in a sea wall for fleet protection, making it a safe harbor. It is questionable as to its affording any protection to the entrance of the Mediterranean Sea. Certainly, it is not the Dardanelles. Beautiful are the surroundings of the city that nestles on the west side of this rock, the mean temperature being such as to allow fruits and flowers to flourish continuously, rendering to the eye of the visitor an ever-changing feast coupled with ancient and grand scenery. The streets abound in soldiers (garrison about 6,000), Moors, Arabs, Spanish, French, Italians, and the omnipresent, industrious Yankee; and it is quite certain the latter is not there for his health. The stores abound in articles of merchandise made in London, Paris and New York. The entire population are consumers.

Here it was that we had the pleasure of an introduction to our most excellent guide, Mr. F. Piromali, of London. Fortunate are those to whose party he is assigned. Not for a single moment did he forget us, and at no time were we found wanting for anything that was possible, as nothing seemed too good for us to enjoy. We now found that characteristic of the people, which seemed to obtain throughout Spain,—"manana",—no one seemed to be after the "worm". "Half-past nine, Sir"!!! This was the time we were asked to be again at the dock with our hand luggage (how I hate that name!) ; of course being Americans we were on time, and after standing (station house 4x6 feet) on the stone wharf for an hour and a half the nice little steamer took our party, now numbering about 50, over about five miles to the beautiful "Emerald Isle" of the Moors,—Algeciras,—the "Key of Spain".

This quiet little place seems to have been a bone of contention. as it was several times besieged,—taken, razed, and retaken,—by Moors, Alfonso XI., Mahomet II., and then Charles III., in 1760, by whom the watch tower on the plaza was constructed and used for observing the movements of his friend (?) the Briton. Just think of George, from the Moorish Castle, and Charles, from his watchtower, playing peek-a-boo, each watching to see that not one grain of sand blew too far on the neutral ground. Here we first came to observe the Roman Arch, in the construction of bridges and viaducts, although now very, very old, still perfect; the streets all paved with cobble stones and no passing vehicles; sidewalks very narrow; buildings small, all white, stone, rubble and Roman brick, covered with stucco; lower windows latticed with half-inch wrought iron "rajas", upper windows and doors balconied; not a sprig of green to be seen on the street, but, when the main entrances of houses (used for man and beast) were open, the court,—patio,—was always fresh looking, with blooming flowers and water. This characteristic obtained throughout the peninsula.

On crossing the bridge, spanning with one arch the old moat surrounding the ancient walled city, we met the ubiquitous beggar with the thickened, granulated, stenosed eyelid, and, of course, being anomalies, we were followed by one or more of the street gamins, these being principally senoritas, who called out with extended arm and open hand. The thought presented itself that here, as in "Twain's Cairo", would be a grand money-making place and country for our enterprising quack nostrum hawker to get rid of all of the Chicago canal water (filtered) as an eyewash instead of feeding the great centennial city with its microbes,—our theory for this prescription being the therapeutics of the future,—"the survival of the fittest". By the way, no American (and right here let me apologise for using the term American as applied to the citizen of the United States, as we found in Madrid the South American delegate very sensitive on this score) can travel in Spain without thinking continually of this great axiom. There is one marked, redeeming feature to all these small bathless residences,—namely, in every window, always upstairs, frequently on first floor, could be seen beautiful flowers. Heaven only knows what the blood-thirsty Spaniards would have been were it not for these softeners of human nature.

This place, in fact all of Andalusia, is celebrated for its beautiful women and oranges. As strangers, we can say that we ate with relish the latter, and did not have an opportunity of seeing the former. The Spanish woman has a sad face; she is undoubtedly a secondary consideration; a necessity not a luxury, and she knows it well. Here as elsewhere, in city and country, except Madrid and Granada, we found the poor, obedient, self-sacrificing, sober, sure-footed donkey, the beast of burden. He carries not only men (the women walk), but every other commodity that has to be transported,—namely, lumber, furniture, water, vegetables, dry goods, groceries, etc.; frequently under the hay pile one would have to look well to find the source of motion.

Here again we found a city of consumers, and the little shops, 6 x 8, replete with articles manufactured in our own country. Our good guide gave us, at the beautiful little hotel, a most delicious lunch at 1 P. M. After this we found in something of a hurry, following Dr. H., our comfort hunter, our first-class compartment, en route for our next stop, Ronda. On reaching the first station out the gendarme, well uniformed and armed with mauser and sword bayonet, was the conspicuous figure. His presence seems to be necessary,—or was more particularly in the past,—as the peasant and gipsy are not owners of land; hence, not positive integers, having very little or no national pride. These armed officials, too, are a necessary evil, even at present, as were not some of them (there being over 300,000 in the empire) ever present the Bandilli would soon demonstrate the actual necessity of the feudal system as in the past. These outlaws seem to have infested Spain until within the past few years.

### EN ROUTE.

Our compartment was a comfortable one for this country, as we were blessed with a lavatory. The compartment would accommodate eight persons, but as soon as our party of five was installed our fumas began to give forth volumes of smoke, and, of course, no one else cared to come in. No bells or pilot on the engine and no conductor,—remember that this is certainly the best way on earth to travel,—that is, to have some one else, Cook & Son for example, take all care and responsibility,—always let the other man work,—especially as no checks are given for luggage. The railroad is certainly a well-laid one, and, although, a very light rail (50 lbs.) is used, it is heavy enough for the coaches. The trains are all mixed, freight and passenger. Our route was, at first, through a slightly undulating country, the track hedged on either side by century plants, making a most formidable fence, with every foot of available soil under the highest cultivation,—oats, peas, wheat, grapes,—orchards upon orchards, as far as the eye could reach, of beautiful olives and forests of cork; the landscape dotted here and there with a little white house and yard full of flowers; the wide pastures, rich with clover and grass, covered by Andalusian cattle, fat and sleek; on the mountain side and again on the plain, the shepherd, with his long staff and highly colored blanket, always folded and thrown over his left shoulder, and the faithful dog, could always be seen.

As we approached Gaucin the speed of the train is checked by the grade and we are reminded of the approach of tunnels by some attachee tramping along on top of our coach, lighting an oil lamp in the ceiling of our apartment. The scenery is now that of chasms, gulches, mountains with small streams dashing along; out from tunnel to chasm and again the trout brook. At 9:20 P. M. we are again at our stop for the night,—Ronda.

### RONDA.

This city is 2,500 feet above the sea level, is built on a rock of limestone, this being porous no dampness follows the heavy rains. This place seems to partake of the Moor, Roman and more recent Spanish. A few minutes walk from the station brings you to an enormous gulch, a dividing line (called "Tajo"), 200 feet wide and 300 to 400 feet deep, spanned by two rock bridges, one Roman, the other Spanish, the latter not more than 150 years old and the one arch at least 150 feet span. The river Gaudalevin rushes along at the bottom of this chasm with force enough to furnish all the power necessary for the flour mills, electric plant, etc. The oldest bull ring in the Empire is here built over the old Roman Amphitheatre, and here we see the remains of an old Moorish castle, showing a recognition of this as an important defensive point.

We would not leave Ronda without speaking of the wonderfully preserved remains of the immense, long Roman Aqueduct, which undoubtedly supplied the city with water. The arches are seemingly as strong and well preserved as if only a few years old.

About 9.30 A. M. (21) our train left for Seville, via Bobadilla. The entire route was at first through mountains then beautiful fertile plains, abounding in orange and olive groves and pastures, some of which were marshy, and here for the first time we had the pleasure of seeing that bird of beauty and grace, the stork, and an occasional pair of handsome quail pheasants, these having bright plumage.

En route we passed through Utrera. This place has been, in the long past, one of great importance, as the remains of old Moorish castles, some of which must have been immense structures, the old cathedral, magnificent in every way, testify; the traditional one owning the only piece of silver left of the thirty that Judas received—the feudal walls surrounding all. From here to Seville, almost all the way, our pathway lay through a valley, strewn with flowers, oranges and olives. Occasionally away to our left, surmounting the high rock on the opposite side of the Guadalquiver river, could be seen the ruins of old castles and monasteries. About 7 p. m. we were in Seville, taken at once by our guide (C. & S.) to the Hotel Madrid and given the very best accommodations that one could ask. The "barber of Seville" was found after dinner. He was clever with his razor, but oh! the chair! you can only appreciate this and the basin (with a scoop out of one side) of cold water after you have once used them. As a sign, the red and white pole is not used, but the basin with concave bite. Our American party went out in the rain to see the beautiful senoritas (De Amices) gracefully trip the light fantastic, with that subtle air of mystery, under an intoxicating Oriental atmosphere, saturated with the fragrance of the orange blossom, the vision feasting on forms more exquisite than Venus de Medici. We expected our hearts to be filled with a delicious sense of amorous delight and melancholy. We went, we saw, we retraced our steps!!!

Next day we visited the cathedral, one of the largest and most magnificent in the world, erected on the site of the old Moorish temple. This immense structure is admired by some of the most celebrated artists the world has ever known, and as you turn your eyes upwards, the gaze meets the most beautiful stained glass of all Europe, as clear and distinct as in 1100. The hour to visit the cathedral is at the Ave Maria, when the sun is low and its rays tremble on the walls in irises of gold; when the great painted windows stand out in a pale light alive with venerable forms of lawgivers, prophets and kings; the delicate curves of the arches melt into dim lines, and rays of yellow light pierce in like arrows upon a burnished ground; then the sculptured saints seem to take form and live, the flying pipes of the organs to glitter like angels' wings, the statues on the choir to murmur in a strange tongue; the many pictures which line the lower walls, to grow terrible in the half light; the sculptured forms of archbishops, long laid to rest in the repose of painted shrines, beside which deacons keep watch with silver croziers, to move; and, from the boundless gloom, aloft to fretted roofs, a burst of sound sweeps like an earthquake; round, harmonious thunders roll, and deep-mouthed pipes speak with deafening shout; the giant organs (two in number) replying to each other as in dialogue of Titians; the human voice, exquisitely sweet, the rattle of triumphant drums, the shrill bray of trumpets, the clash and clamor as of a battle field; then the loud anthem ceases as suddenly as it began. The old ebony clock ticks out again from its dark frame; the dull drone of chanting priests uprises from the choir, and the silent worshippers creep in and kneel.

On the southeastern corner of this immense building we find the watch tower (called Giralda), erected by the Moors, in 1196, as an observatory, the first on the continent; this towers far above the dizzy height of the old cathedral and covers quite a large area, as one could drive to the top by its broad winding road. The outside is beautifully ornamented by the Moors, to the upper tier, where are found the bells and cupolas, added by the Spanish, in 1568; changed thus into a belfry as the Spanish did not know its purposes. From the summit you have a grand view of this city of 400,000 souls.

The Alcazar, or house of Cesar, is certainly a well preserved castle, used for years by the Moors and since by the catholic kings. Alfonso "our boy", as the guide said, has his rooms here when visiting the old seat of government. The "Hall of the Ambassadors" is the most beautiful of all the dozens of compartments. It is "literally marble floor to mother of pearl and crystal roof one blaze of iris hues". The immense garden is certainly exquisite, the walls with their bright stuccoed paintings, fountains still active, and kiaks all speak the Moor, and every sovereign from Cesar down, has marked this delightful spot.

The streets are narrow, never following straight lines. The houses are rather attractive, light blue, pale green, pale yellow, or pale rose. Here, as elsewhere, the "patios" are a striking feature in the Sevillian houses. As described by one author,—a "patio" is a speciality, half Moorish, half Roman; not a court, nor a garden, nor a hall, yet all three,—being converted when necessary on account of heat, into an eating, sleeping place, or a regal place for entertainment. An open work wrought-iron gate shuts off the intruder, and in the midst of this area or patio is a fountain surrounded by banks of beautiful flowers.

This is certainly the most Oriental of all European cities; and is rendered historic by having given Rome three of its rulers,—Hadrian, Trajan and Theodosius,—the famous voyager Magellan, and the artists, Murillo and Valasquez. It is not only the birth place of the two master painters mentioned, but we find in the museum here, some of their finest works. A facsimile of the house of Pontius Pilate, built by a native of Seville, is one of the most beautiful private residences. The Plaza del Toros, built in 1760, as perfect today as then, accommodates 12,000 spectators. The Torre del Oro (Tower of Gold), an enormous octagonal castle, built on the margin of the river Guadliquiver, for the purpose of receiving gold and trophies brought here by conquering heroes or explorers, and the palace of San Telneo, all constitute places of remarkable interest. On the evening of the 22d, at 7.30 o'clock, we took our sleeper for Madrid, arriving, after a rather comfortable sleep, the morning of the 23d.

THE CONGRESS.

On reaching our hotel, The Santa Cruz, being under the wing of Mr. Piromali, we had no fear in regard to accommodations. At once putting on our fatigue uniforms, we sought the dining-room for breakfast,—11 A. M.,—as en route from Seville, the dining car had only furnished eggs, coffee and rolls. Soon afterward we were directed, not by anyone from our own delegation who should have known something of plans and communicated the same, to the National Art Gallery and Museum, where we could register. I may as well mention here that the total registration and attendance was 6,981, the official delegates from respective nations being 474.

Before Spain was located by geographers, before the first stone of the bull ring was laid at Ronda, far in the Orient, a set of workmen became so confused in the construction of what would have been the most wonderful monument on earth, that all building was discontinued. To say that we found a second Babel on entering the registration room, but feebly expresses it; German, English, in fact, every dialect known was being expounded, not in whispers by any means, by an immense throng of hurrying physicians, seven-tenths of whom could not make themselves intelligible to the Latin at the window or behind the counter. Our credentials when presented were at once pushed back, and we were made to understand by a sign of disgust that these official papers were not necessary; but we were asked for the secretary general's receipt and on producing this we received the badge, program, invitations and all necessary papers. We asked for the bureau of information, but there was none; we asked for the official medical directory, but again, there was none; there were no tickets of admission to the opening exercises; all had been given out to anyone who chose to ask, with or without a badge, physicians or laymen. Let it be understood that we are not complaining, as it was plain to be seen that the committee of arrangements was looking for a purely Latin congress. It was rare during the entire meeting to hear either German or English, and no interpreters were to be found.

Again, at the mass meeting held at the San Carlos Medical College on the morning of the 30th, not one-tenth of the visiting physicians were made aware of it; and it was so every morning at this general conference. Lisbon was chosen as the next place of meeting and the XV. will become practically another Latin congress, making two in succession,—an unfortunate circumstance indeed; but it is to be hoped that this almost sister city will profit by the omissions at Madrid. The hotel accommodations were limited at Madrid; what will be the condition in Lisbon? We hope the secretary-general will see that some space is given to the Germans, English and Americans. There are but few cities that can comfortably lodge 5,000 delegates, especially when one-third more must be added for wives and children.

On the afternoon of the 23d, at 3 o'clock, the formal opening of the XIV. International Medical Congress, by King Alfonso XIII., took place at the Royal Theater in the presence of the entire royal family, and one of the most magnificent audiences of more than ten thousand, made resplendent by the beauty of costumes, flowers, flags and jewels. As the King entered the royal box the palace band struck up the royal march, and at once the entire audience rose to its feet. Alfonso was dressed in a Field Marshal's uniform, everyone also being in full dress, military or civilian,—a magnificently dazzling sight. After listening to the address of welcome from the king by his exponent, as he never speaks in public, the president of the congress, secretary-general and mayor of the city,—each with an address of welcome,—the different nationalities were called upon to reply; and, when our beloved country was called, no one responded,—a want of organisation on the part of our delegation, or delegate supposed to be the organiser, if not the mouthpiece of the body. 'Tis an ill wind that blows no good, and the Spaniards seemed to think this a beautiful piece of courtesy on the part of the United States. In the evening at 10 o'clock, the mayor's palace was thrown open and a most beautiful reception was given. Here, again, the host failed to reckon on his guests; the tickets were short.

On the morning of the 24th, at 9:30, the congress, in 16 sections, came together in the different salons of the National Art Gallery and Museum, and unfortunately, for three reasons, these commodious halls were anything but working places for a scientific body: first, the walls were all hung, and every available inch covered, with masterpieces of painting (not considered so here because of want of age); hence, diverting, if not to the Castilian, surely to the Anglo-Saxon; second, the doors of the institution were always open to the sight-seers, many of these of our own profession, who were constantly walking through from one salon to the other; third, there being no doors, the speakers in the next sections, right and left, could be heard, and the applause was frequently in the wrong place. The number of communications by title were 1,681.

In fact there were nearly 2,000 titles of papers sent in to the secretary-general, so it will be observed there was no lack of material for "neuron" exercise. The time allowed each paper was 20 minutes, and discussions were limited to 5 minutes. The great majority of valuable papers were read and discussed in Spanish, and, if there is one thing (besides wine, women, song and the bull ring) that the Spaniard is fond of it is discussion; and, although the entire discussion was unintelligible to most of us, there is no keener thrust you can give a Castilian than to get up with the American rudeness and leave the hall while he is speaking. As usual, a great many of the American "title writers" were conspicuous by their absence when called; this is rather characteristic of our countrymen. Again, quite a number "turned the barrel over" and reread from printed monographs.

Spain will astonish the medical man. The kingdom abounds in up-to-date searchers for medical and surgical truths. There is no doubt but that there was too much sightseeing, hence the sections were not as well attended as they should have been.

The Castilian is certainly the grandest of hosts. On the afternoon of the 24th, at 3 o'clock, a magnificent, full-dress reception of welcome by the king was given at the Royal Palace. The entire congress was passed in review by Alfonso, the Queen Regent, the Infanta and Infanta Isabel, each one, in turn, stopping and talking with two, three or more of each delegation. Ours, headed by Surgeon General O'Reilly, U. S. Army, and Colonel Senn, cared for and introduced by our bright and genial minister, Mr. Hardy, assuredly compared well with the others, as was evidenced by each of the royal party delaying so long and talking with quite a number of our delegates.

The young king is certainly very bright and much of a man, and we were all favorably impressed by his noble and unassuming manner, he even speaking cheerfully to the Cuban delegation, which was on our immediate left. The Queen Regent (Marie Christiana,—the Austrian) did not stop or speak to our neighbors. The Infanta has a strong, kind face, markedly unassuming, goes and lives with and is worshipped by all Spaniards; she is a true Mercedes. After the reception we were asked to the patio, and here had a feast for the eyes, in the $10,000,000 worth of most beautiful tapestry, the largest collection in Europe. It is useless for one to attempt a description of this beautiful palace. Friday night we were more than satisfied to rest, but we felt as though we were losing a great deal, as Madrid does not get awake until 11 o'clock P. M.

Saturday, the 25th was spent at the military section, from 9 to 12. In the afternoon we were given a drive out five miles to the Hospital Militar, and after visiting almost numberless "units", operating rooms, laboratories, supply rooms, etc., were highly entertained at a royal luncheon, followed by a most excellent hospital corps and ambulance drill, returning to the hotel about 6 P. M.; dinner and then again in full-dress uniform, we whirled off in carriages to the most princely reception, given by the Secretary of State. This reception was one of the most beautiful we have ever seen or attended. It would have been impossible for a Cesar in Rome's golden age to have entertained with more magnificence. Sunday morning the 26th, after our eggs, coffee and roll, we strolled to the old church, made famous by Isabella II., and at 10, crossing the Prada, entered the Gallery of Art.

Monday, 27th, we attended the Military Surgical Section at 9. Of the papers read in this section only one,—"The problem of tuburculosis in armies", by Stricker, of Berlin,—occupied the attention of the section, it being replete with interest. At 1 P. M. an elaborate luncheon was given by the medical officers of Madrid to the section, and the afternoon was spent in the National University and Chemical Laboratory; here all the medical supplies are made for the army and navy. In the evening the section was invited to attend the opera, at the Theater Lyric.

Tuesday, April 28th, 9 A. M., section was called to order, Colonel N. Senn in the chair. We were called on for our papers, after which Colonel Senn read and demonstrated his new emergency package, characteristic in its simplicity and excellence and ease of application, being the best so far for use, and its adoption in our army should follow. Next, the colonel presented his new, beautiful, efficient and most thoroughly compact operating case, one of which every regiment in the U. S. Army should be supplied with, not larger than the old cartridge box, and instruments, in canvas, with extra folder ready for throwing into boiling water, and containing every instrument necessary for any operative procedure. This case he presented to the Navy of Spain. The afternoon was spent in visiting the Public Hospital and the Naval Museum and Hospital, having been invited and accompanied by Capt. John Redoudo, of the Navy,—a man and a true soldier. This beautiful hospital is located north and west of the city, just outside of the old city walls, and on an elevation just to the right of the entrance to the Royal Park, and from here you have a most beautiful view of the capitol. Colonel Senn, in the evening, entertained our good friend and Madris, Salvator, Capt. Redoudo, at a beautiful dinner.

April 29th, we had a good morning's section work, followed by a beautiful farewell luncheon and short speeches. In the afternoon the royal family entertained the congress at a most beautiful garden party in the palace gardens. A most delicious luncheon was served in the open, and champagne was a great deal freer than water. The King, his mother, aunt and sister were driven up in the royal carriage and alighted to the strains of six bands of music, one as fine as I ever heard, composed of 63 professors of the art. King Alfonso mingled everywhere in the throng of guests. The evening was spent by a great many of the delegates at the residence of our minister. April 30th, the general conference was held at San Carlos College of Medicine, and Dr. Howard Kelly, of Johns Hopkins, delivered the address for the United States. The sections then followed with the election of officers for the next congress, to be held at Lisbon.

The XIV. International Medical Congress is now numbered among the things that were, and is a matter of history. Let the good that men do live after them; let's bury the evils, looking upon the sins as those not of commission but purely omission.

The committees in charge have undertaken and accomplished satisfactorily to a great extent an Herculean task, and we thank them one and all, from the bottom of our hearts.

A few suggestions, however, we hope may not be out of the way. The rules for membership are entirely too lax; delegates should emanate from national organisations; the American Medical Association and the Association of Military Surgeons, are the only two bodies in the United States that should appoint delegates, and these should be sent in the proportion of one to one hundred members. Moreover, the official delegates alone should be allowed on the floors of the general assembly or sections. This, if attended to by properly appointed committees, will give to the delegation a standing and organisation, neither of which was attained at Madrid. The government should appoint from the Association of Military Surgeons official delegates. No layman should be allowed to register; no one should be allowed to register without filing with the secretary-general his papers or certificates from delegating body, these to be kept in the archives of the congress.

### MADRID.

On account of its rarified air and interior location Charles V. was induced to vacate the older capitals,—Seville, Toledo and

Valladolid,—and fix his royal residence here, on the wooded cliff, high above the valley of the Manzanares. The city is not as markedly Spanish in appearance as the others of the empire. The streets are wider and the larger shops to a great extent resemble those of Paris. Large squares abound, planted with trees, ornamented with flowers and fountains. All of the large, fine buildings of the city were erected by Charles V. When the fires of the inquisition were lighted, the old gateway, then the principal one, Puerto del Sol, was located where now ten large Calles converge in the very midst of the city. This open square is where the fevered pulse of Madrid beats the fastest; though not even handsome, here you see and meet everybody; knock up against a Minister of State; brush against a well-shoed "torero", with his chignon of black hair, short jacket and majo hat; tumble across an ambassador or a match tray, or hitch yourself into the linen veil of a sister of charity, book in hand, coming from mass; jostle an estudiante, or a Castilian peasant in breeches, smelling of garlic, or face the domestic cook, with his basket of provisions poised on his head. At the Puerto del Sol merchant meets merchant, politicians scheme and demagogues plot. Here pronounciamentos are planned and the jeunesse doree gamble. The Puerto del Sol is the place to look for a friend, to avoid an enemy, hide from a creditor and a rendezvous for mistress or lover. If you are jealous you can stab your rival there, or be in turn yourself turned off by a blade; the crowd, like charity, covers all.

The royal palace, the museo and the armory, stand first in their classes in the world. The bull ring, containing the royal box, is the largest extant, seating 22,000 comfortably. The royal palace is one of the most magnificent in the world; the salons are beautifully furnished and the hall of the ambassadors is princely with its decorations; the ceiling painted by Tiepalo, "the majesty of Spain." It was in this room we were received. The picture gallery can boast of 62 Rubens, 53 Teniers, 10 Raphaels, 46 Murillos, 64 Velasquezes, 22 Van Dykes, 43 Titians, 34 Tintorettes, 25 Veroneses, 54 Breughels, 23 Snyders, and myriads of others. It is useless to speak of delight and bewilderment. One wishes for weeks of time. The royal armory was established in 1565. The collection is considered the most complete in the world, and the pieces have belonged to many of the most renowned warriors in medieval and modern history. What a sight! You rub your eyes as you find yourself in the company of the Cid Campedore, Ponce de Leon, Boabdil, St. Ferdinand, James the Wise, the great Captain Gonzalo de Cordova, Columbus and Charles V. The walls are sheeted with guns, swords, halberds, lances of tourney and battle, muskets, pikes and cross-bows, each with a history; banners, trophies from Lepanto, St. Quentin, Naples, Mexico and Peru, from Cuba and the southern states; armed and armored effigies of all the early kings; Charles V. in the same massive armor in which Titian painted him at the battle of Muhlberg, with the same crimson panache on his helmet; Philip, his son, in the same armor, inlaid with gold and silver, the arms of England on his breast as painted by Titian; Philip studded with cameos and Philip in silver filagree; Ferdinando Cortes, who came to his death, forgotten, near Seville, in the curiass he wore in Mexico; Columbus in plain black and white suit; Hannibal and Cesar; the visor and jewelled sword of Isabel, the catholic, as she appeared before the walls of Alhambra; the halberd of Peter, the Cruel; the dagger of Pizarro in its steel sheath; the darendana of Roland, the Brave, upon which he spitted his enemies like larks; the immense broad-sword of Gonzala; two magnificent shields, chiseled, representing the Rape of the Lock.

### THE BULL RING.

On Sunday afternoon, April 26th, at 4, we found ourselves on the stone seat of the bull ring, an immense amphitheater, seating with ease 22,000, and not a vacant seat, except in the royal box, this being occupied by only one person, Infanta Isabel, the Mercedes. This is one place where the Spaniard is on time, and nothing must interfere with the slaughter. This was evidenced by the fact that this was election day throughout the city, if not the empire, yet from 6.30 A. M. to 3.30 P. M. everybody was for the bull fight. Our experience will last in memory; the multitude of folk, eager and excited, making their way along Calle Alcala, all seemingly emanating from the Puerto del Sol; all rushing to one place, aroused as nothing but the prospect of blood can stir them. And to stand and look down upon the seething crowd of 22,000 souls, all of one mind and desire, from the elegantly dressed senoritas and cavaliers, down to those who, perhaps, have had to pawn their shirts to be there!

This Spanish crowd has well been called seething; however, there was no boiling over, no riotousness, no one drunk, as the native drinks wine, is never boisterous, and "never full but just aplenty". In fact, it is astounding how decorous this huge mass of humanity is awaiting the longed for event. The president enters his palco or stage box, the signal is given, and forth comes the Candrilla. This opening ceremony is pretty and effective, headed as the procession is by a couple of caballeros in black velvet, riding black steeds; next two espados, one in yellow and violet and the other in gold and green, these being the heroes of the day; next half a dozen picadores in short cloaks and leather leggings, plated with steel; now come the banderilleros, with bright silk sashes, short breeches and highly colored stockings; and the last of the procession, made up of four or five attendants leading mules, bedecked with plumes and trappings,—these for the purpose of dragging off the carcasses from the arena. The entire group now moves across the arena, salutes the president and breaks up. The picadores canter away taking up their respective positions at various points; the banderilleros find their red cloths; two cabelleros, that seem to be left behind, salute the president and the keys of the bull's den are thrown by the latter to them. Now, in a very few moments the band ceases its lively music and the first bull dashes into the arena, as though shot by a catapult.

THE FIGHT.

This masterpiece of brute life at first seems bewildered, rushing to right and left, chasing his tormentors over the barrier, and after seemingly clearing the arena of pedestrians, rapidly turns his attention to one of the picadores, mounted on a poor, unsuspecting blind-folded horse; and, although the horseman does his utmost, with a long pike, to turn his assailant aside, on he comes with lowered head, a peculiar digging operation, then a momentary upheaving and down goes the horse and man, apparently used up, if not dead. Now, the banderilleros divert the tortured animal by their red cloaks, thus giving the picador an opportunity to escape. Again, a shout is heard, the horse is helped up, and, although disemboweled, yet the picador mounts and the poor animal is lashed onward for another and, as we hoped, final assault. Unfortunately the picador has escaped without even being crushed by the horse's fall.

In a few moments the bull starts for one of the red cloak gentry, following closely over the barrier which he leaped, the bull clearing the same immediately afterward. Now the excitement runs high, and the ether is rent with wild cheers, mingled with hushed hisses. All the picadores have had their turn; or maybe the thirst for blood by the spectators has been satisfied for the time being. The president gives the signal and the picadores retire. The banderilleros enter the arena, their office being to stick into the shoulders of the bull three or four pairs of banderillas, which are ornamented darts, about two feet long, well barbed and exquisitely pointed, indeed, often very valuable.

This is a pretty sight to witness, if any part of the bloody orgie could be so considered, requiring great skill, agility and daring. The principal stands in front of the bull, and raises his arms as high over his head as possible, in a very defying manner. He then walks slowly forward, the bull makes a rush, and the man quickly dodging to one side, pins in his banderillas simultaneously. Now there is a burst of applause if the effort is crowned with success; if, however, there is bungling or evidence of fright, he is unmercifully hooted and hissed. These performers become very much aroused by the voice of the multitude and vie in feats of daring. One will take his seat in a chair as the animal makes his maddened rush, jumping lightly up as the horns of the animal graze by, manipulating his darts in an unerring way that seems almost impossible; another, with wonderful nicety and precision, leaps clean over the brute's lowered head; another will seize the bull's flying tail, and swinging plant himself for a moment between the curved horns. The cruelty of the people not being satisfied with this torture, the cry of "fuego", "fuego", is raised and the banderilleros are at once provided with firework banderillas, half squib, half cracker, which, hissing and exploding in the side of the poor beast, is certainly the ne plus ultra of fiendishness.

Now the signal sounds again, and *The Actor*, the principal espada, steps out, greeted with wild applause; the one to finish the weird, bloody, strange scene, with his short Toledo blade and a red cloth. He walks forward to the bull with the nervous action of an athlete or fencer. Now the tumultuous throng is impressively hushed, eyeing closely every movement of both man and beast. The espada must play his victim in an approved fashion. Everyone now sees that the bull is markedly exhausted, still he is all pluck and makes head against his enemy, who, aided by his squadron of chulos, resorts to every method of irritation and punishment. Suddenly he stops, within a couple of feet, face to face with the fascinated animal; the man is now, evidently, on exquisite tension for a moment,—then the sword is buried to the hilt in the brute's neck between the shoulders. The one blow finishes the horrid spectacle. The bull staggers down on his knees and then falls heavily to the ground. The attendant now finishes by a stilleto stroke through the medula and the espada, being greeted by deafening cheers, a beautiful bouquet and showers of other tributes, walks toward the president's box. A fiendish conjunction—butchery, flowers.

Now all is over; the band plays; the mules are brought in, harnessed to the carcasses that lie upon the arena and are driven off at a gallop; the sand is raked over the streams and pools of blood; the watering cart lays the dust, and everything is now ready for number two. Six animals and twelve horses were killed during the two hours' performance—but not a single man.

The second and fourth contests were the wildest and most picturesque, the former killing three horses in less than that many minutes and clearing the ring of every living being. This accomplished, he walked completely around the ring, every few paces looking appealingly up at the hissing crowd as though asking —"Have I a friend anywhere?" The bullfight, thank Heaven, as a diversion, is steadily losing favor with the fair sex, hence its blotting out is assured.

### GRANADA AND THE ALHAMBRA.

Leaving Cordova on the afternoon of the 1st of May, the same evening found our train in the station at Granada, a city of 75,000 people. To the tourist probably this is the most interesting city in Spain, situated on a lovely plain, 2,250 feet above the sea level. We were driven across the city in an easterly direction through the Puerta Real into the grounds of the Alhambra, traversing a winding boulevard, on either side of which we could hear, above the noise of the wagon and lashings of the driver, the rushing waters. A little farther, and on our left, towering against the star-lit sky, we could distinguish massive turreted walls. This was Irving's Alhambra. A few moments more and our vehicle pulled up at the very door of the Washington Irving, a nice, clean hotel, beautifully located on either side of the boulevard. Being well tired out we sought our rooms for sleep, certain that our dreams would be spiced with visions of adjacent wonders. On awakening we found our window overlooked the walls of the Alhambra and Irving's room, where the author lived for ninety days.

At nine next morning we were following our guide, and en route to the Puerta del Justica, where we were surprised by seeing several streams pouring out from the walls through lead pipes, seemingly as good as when placed there in the XV. century. On approaching the gate one is reminded of the hand of Isabel, the Catholic, by the niche in the stone, just above the keystone of the Roman arch, in it placed the statue of the Holy Mother, and on either side niches filled with blooming flowers. The great swinging doors, 94 inches in thickness, of hardwood and iron interlacing, give fruitful evidence of the stubbornness of the garrison and vigorous use by the besiegers of catapult and crude cannon ball; however, this was not the place of entrance of the conqueror Charles's force, it having made a breach in the southeast bastion on the ground level just opposite the corner of our hotel.

The pride of Granada and the boast of Spain is the Alhambra, the ancient palace of the Moorish kings. The magnificent ornamentations of this celebrated time have made it an everlasting memorial of the talent and exquisite taste of its builders; it stands unrivaled, defying pen pictures, even by Irving, in the gorgeous splendor of its halls, and up to this date its decorative art has never been paralleled,—we can only say: enjoy with Arabian Nights, fantastical mental creations; proceed with ever-increasing and appalling wonder, from the Puerta delos Carras across the great square to the modern entrance, thence to the Patio of the Fish Pond, .Court of the Lions, Hall of the Abencerrages, Hall of Tribune, Hall of the Two Sisters, Boudoir of the Sultan Lindaraxa, overlooking a beautiful garden, Moorish baths, Hall of the Camas, the resplendent Hall of the Ambassadors; Halls of the Palace of the Walies, ancient chapel, with the Koran now the Host facing the east. Next we visited the Palace of Charles the V. and across the great square to the Tower of the Vela, or watch, with its small portals, off from the winding, well worn, marble steps, leading to halls of Calcutta. From its summit is a magnificent view of the plain of Granada with the city at your feet, bisected by the Darro and Genil rivers, the yards resplendent with every tropical fruit while, in turning around, your eye meets the snow and glaciers of the Sierra Nevada mountains.

Under the Moors, Granada numbered 500,000 inhabitants. The houses of the old city looked markedly eastern, many of these being brilliantly painted, and the streets narrow and winding. In the beautiful cathedral is the Capilla de los Reyes, the burying place of Ferdinand and Isabella and the remains of Joan of Arc. These sepulchres are the most magnificent in the world.

We left this beautiful place on May 3d at 8.30, wishing for a stay of at least two weeks or more, and for 50 miles no one spoke, except to ask for a match to enable a stronger pull at his cigar,— all absorbed in thought. We arrived at Algeciras at 10 o'clock P. M., then crossed to Gibraltar, went to the hotel and were soon asleep, dreaming of our start for home, as we were to sail next morning.

GRAND AVE. AND 35TH STREET.

